# Supplementary figures and images for: A novel cancer immunotherapy using tumor-infiltrating B cells in the APCmin/+ mouse model
Source: PLoS One. 2021 Jan 19;16(1):e0245608. doi: 10.1371/journal.pone.0245608 (PMC7815094; doi:10.1371/journal.pone.0245608)

IgA

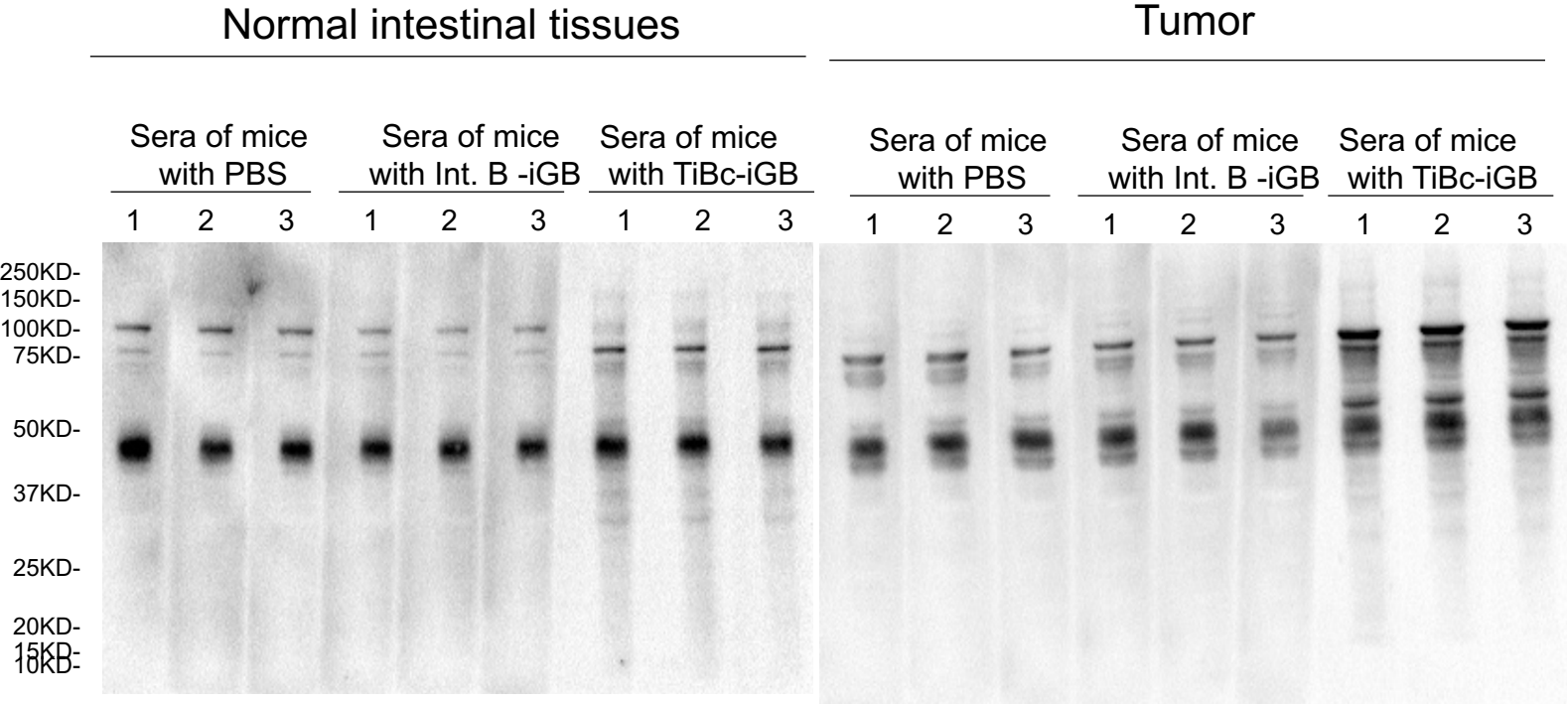

IgM

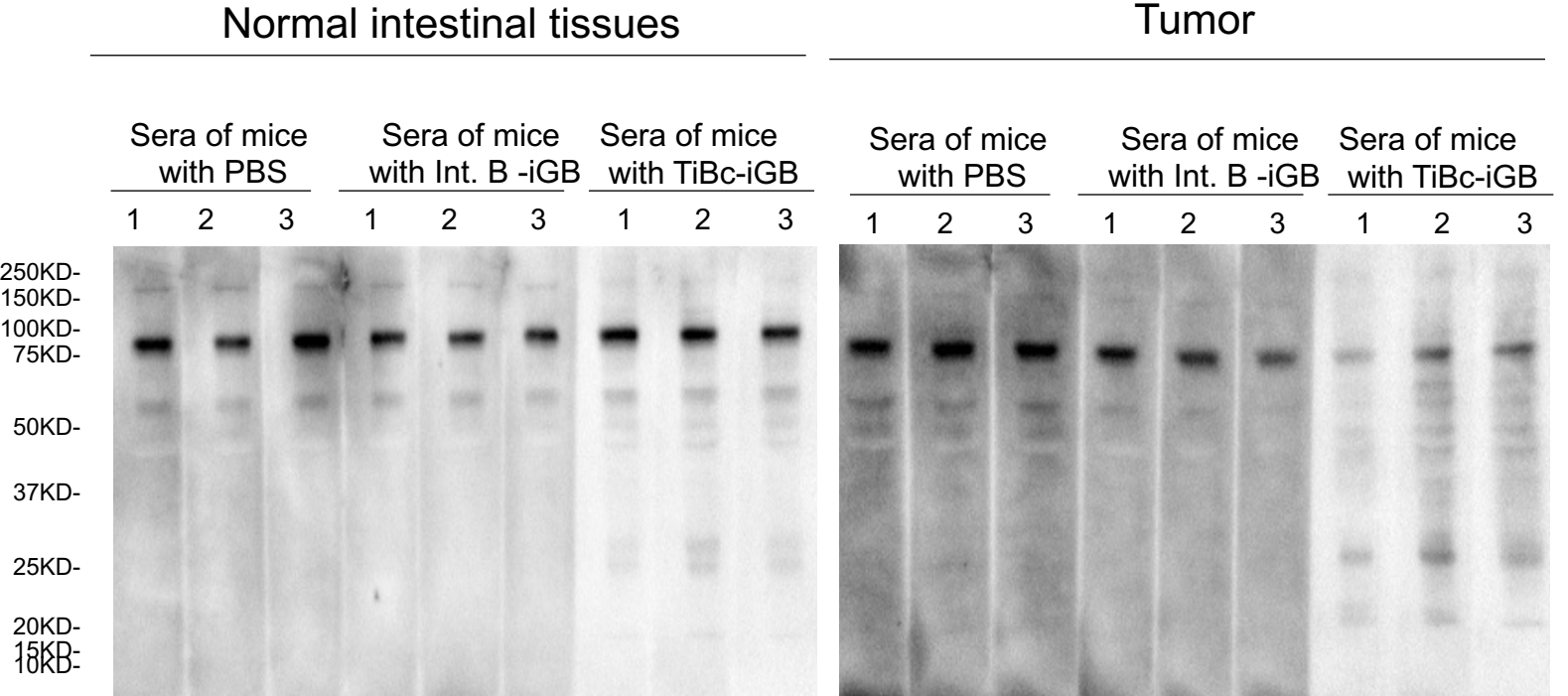

Supplement: S1 Raw images — (PDF) [file pone.0245608.s002.pdf]
