# Supplementary material for: A novel cancer immunotherapy using tumor-infiltrating B cells in the APCmin/+ mouse model
Source: PLoS One. 2021 Jan 19;16(1):e0245608. doi: 10.1371/journal.pone.0245608 (PMC7815094; doi:10.1371/journal.pone.0245608)
Supplement: S1 File — (ZIP) [file pone.0245608.s003.zip › WB IgG original/GAPDH.pdf]

图像报告: Bio-Rad 2020-10-23 18hr 46min_Exposure_1.9sec

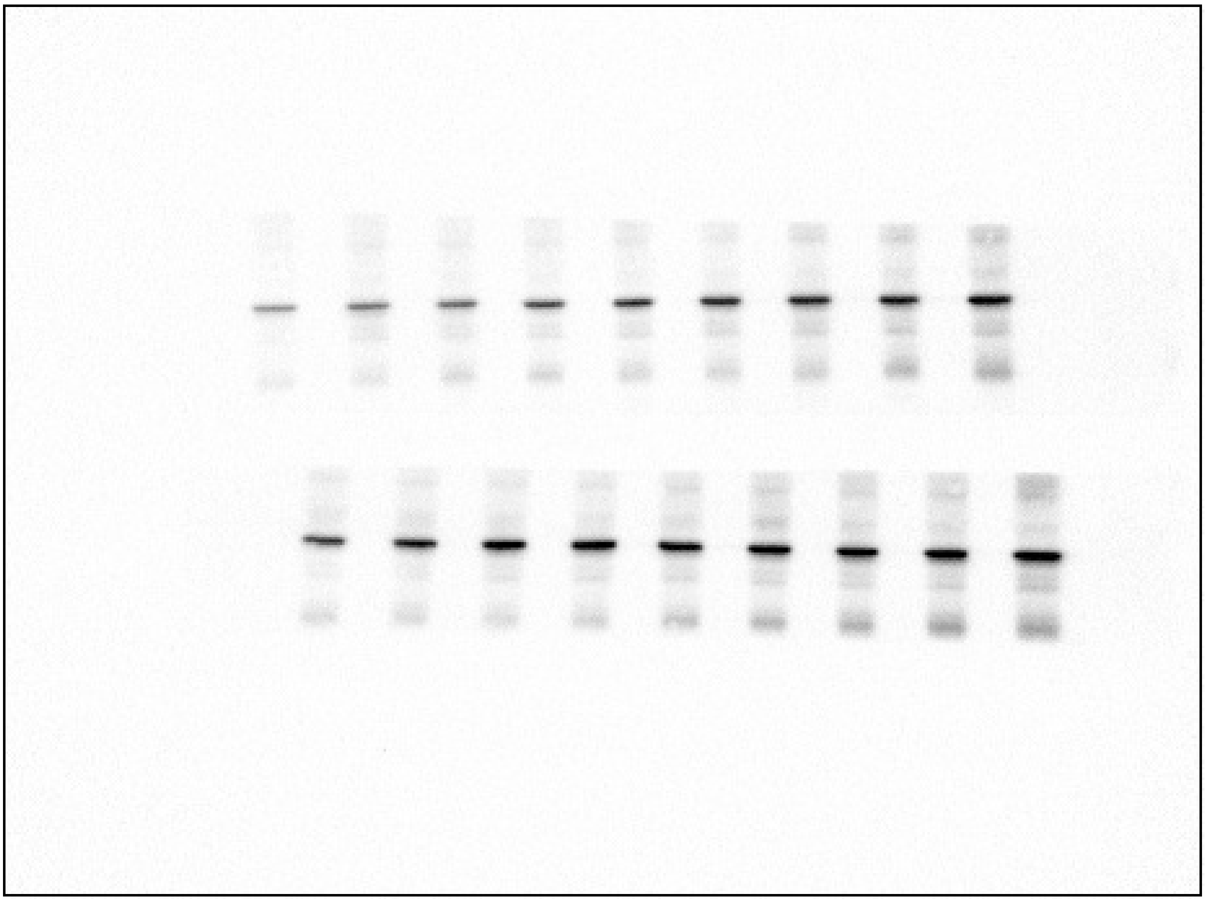

D:\实验数据-博\实验结果\APC鼠\Ag WB\201023\GAPDH\Bio-Rad 2020-10-23 18hr 46min_Exposure_1.9sec.scn

获取信息

成像仪	ChemiDoc™ MP
曝光时间(秒)	1.888 (信号累积)
平场	Applied (Lens)
序列号	731BR03129
软件版本	5.2.1
应用程序	Chemi
激发源	No Illumination
发射滤光片	No Filter
像素组合	3x3

图像信息

获取日期	2020/10/23 18:46:15
用户名	Bio-Rad
图像区域(mm)	X: 165.0 Y: 123.0
像素大小(um)	X: 355.6 Y: 355.6
数据范围(Int)	0 - 33580

分析设置

未执行分析
