# Supplementary material for: A novel cancer immunotherapy using tumor-infiltrating B cells in the APCmin/+ mouse model
Source: PLoS One. 2021 Jan 19;16(1):e0245608. doi: 10.1371/journal.pone.0245608 (PMC7815094; doi:10.1371/journal.pone.0245608)
Supplement: S1 File — (ZIP) [file pone.0245608.s003.zip › WB IgG original/IgG Normal intestinal tissues.pdf]

图像报告: IgG Normal intestinal tissues

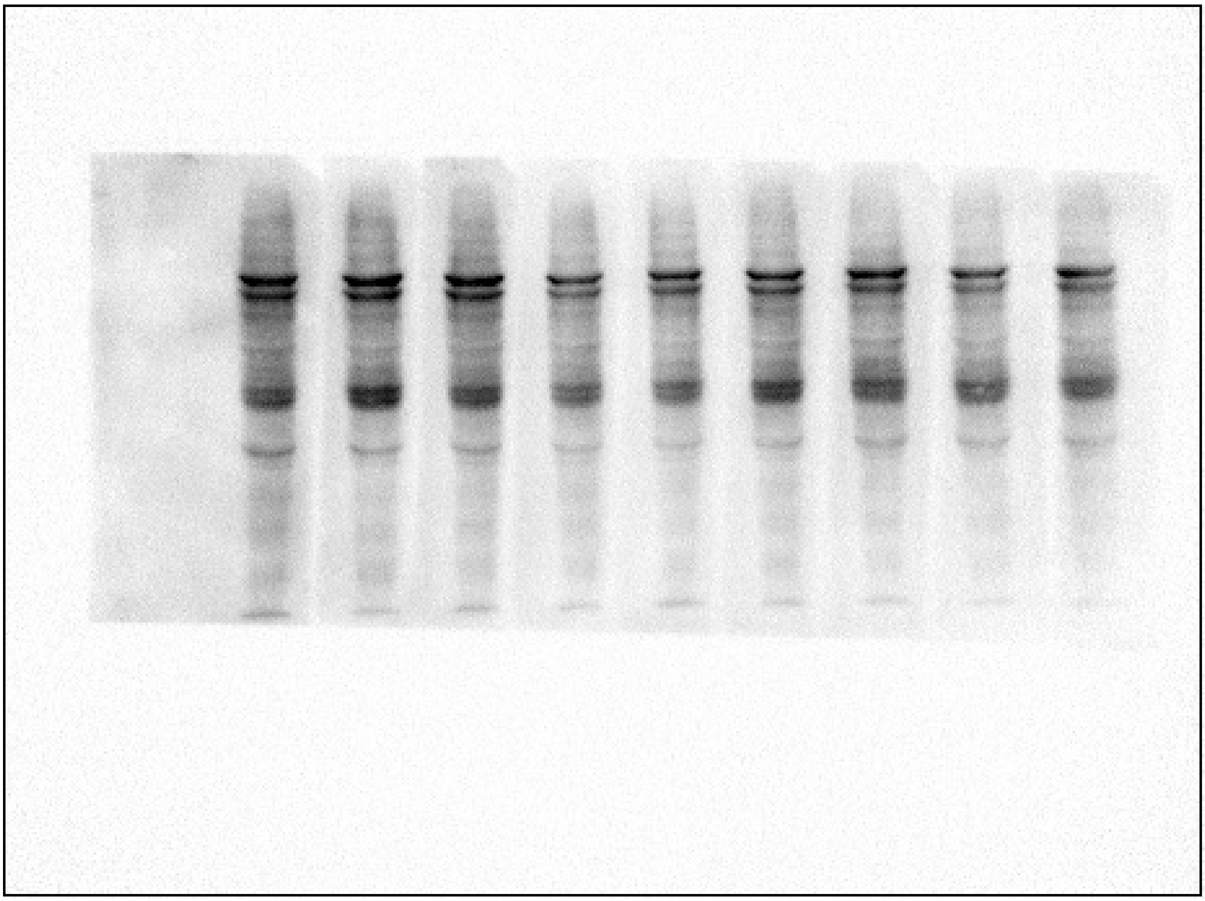

C:\Users\l\Dropbox\My PC (LAPTOP-PUBHJFH9)\Desktop\WB\IgG Normal intestinal tissues.scn

获取信息

成像仪	ChemiDoc™ MP
曝光时间(秒)	4.311 (信号累积)
平场	Applied (Lens)
序列号	731BR03129
软件版本	5.2.1
应用程序	Chemi
激发源	No Illumination
发射滤光片	No Filter
像素组合	3x3

图像信息

获取日期	2020/10/6 13:28:21
用户名	Bio-Rad
图像区域(mm)	X: 145.0 Y: 108.1
像素大小(um)	X: 312.5 Y: 312.5
数据范围(Int)	0 - 39028

分析设置

未执行分析
